# At least two distinct mechanisms control binocular luster, rivalry, and perceived rotation with contrast and average luminance disparities

**DOI:** 10.1371/journal.pone.0215716

**Published:** 2019-05-21

**Authors:** Richard S. Hetley, Wm Wren Stine

**Affiliations:** Department of Psychology, University of New Hampshire, Durham, NH, United States of America; McGill University, CANADA

## Abstract

When one views a square-wave grating and dichoptically changes the average luminance or contrast of the monocular images, at least three perceptual phenomena might occur. These are the Venetian blind effect, or a perceived rotation of the bars around individual vertical axes; binocular luster, or a perceived shimmering; and binocular rivalry, or an alternating perception between the views of the two eyes. Perception of luster and rivalry occur when the "light bars" in the grating dichoptically straddle the background luminance (one eye’s image has a higher luminance than the background and the other eye’s image has a lower luminance than the background), with little impact from the "dark bars." Perception of rotation, on the other hand, is related to average luminance or contrast disparity, independent of whether or not the "light bars" straddle the background luminance. The patterns for perceived rotation versus binocular luster and binocular rivalry suggest at least two separate mechanisms in the visual system for processing luminance and contrast information over and above their differing physiological states suggested by their different appearances. While luster and rivalry depend directly on the relation between stimuli and the background, perceived rotation depends on the magnitude of the luminance or contrast disparity, as described by the generalized difference model.

## 1. Introduction

Binocular vision allows us to detect differences between the two eyes' views, extracting information from binocular disparities. (We use terminology from Macknik and Martinez-Conde [1]. Binocular image and monocular image refer to an image pair presented to two eyes or an image presented to one eye, respectively. Dichoptic image refers to a binocular image that has a disparity or disparities between its two monocular images. Monoptic image refers to an image that has no disparity. Fused image, a term from general usage, refers to a participant's unified perception of the presentation.) Geometric disparities are perhaps the best understood type of disparity giving rise to stereopsis [2]. Luminance and contrast disparities, where the stimuli viewed by each eye are geometrically identical to one another, can give rise to at least three distinct perceptions depending in part on the geometry of the stimulus.

**Fig 1:**
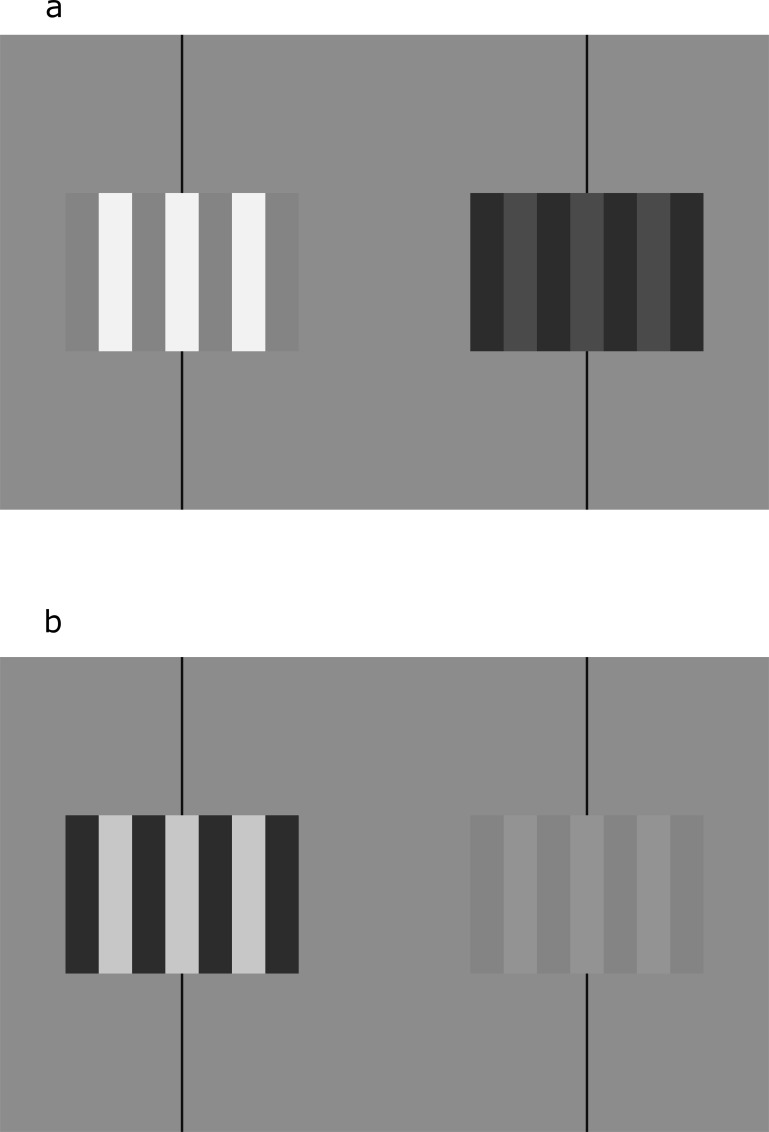
Venetian blind effect examples. Stereograms of rectangular-wave gratings with zero geometric disparity and (a) a luminance disparity corresponding to a dichoptic luminance modulation of approximately 0.8 or (b) a contrast disparity corresponding to a dichoptic contrast modulation of approximately 0.8, for demonstrating the Venetian blind effect. Either crossed or uncrossed fusion is appropriate. If crossed fusion is used, the lighter bars of the fused image will appear to rotate with the left edge of each bar appearing closer to the viewer. If uncrossed fusion is used, the lighter bars of the fused image will appear to rotate with the right edge of each bar closer to the viewer.

One perception was recognized by Münster [3, 4]. Binocularly viewing a pair of geometrically-identical squares with luminance disparities will yield a perception of rotation in depth. Cibis and Haber [5], independently rediscovering perceived rotation due to luminance disparities, coined the term Venetian blind effect. In the Venetian blind effect, viewing a vertical square-wave grating with a luminance disparity (Fig 1) gives rise to a perceived rotation of the bars in the grating around individual vertical axes, as does viewing a square-wave grating with a contrast disparity [6, 7].

**Fig 2:**
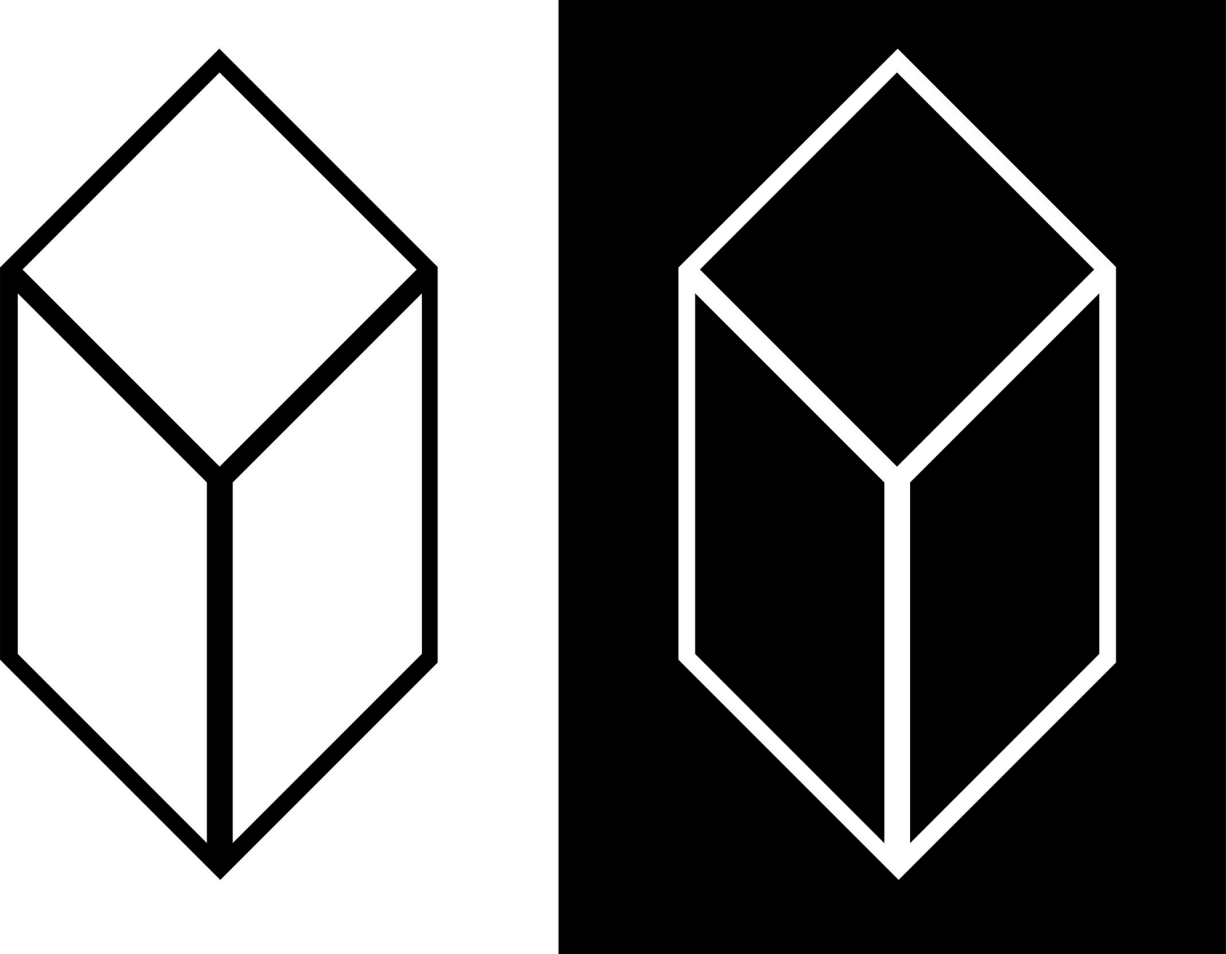
Binocular luster example. Stereogram of "crystal" image with maximum luminance disparity for demonstrating binocular luster, taken after the written description by Helmholtz [9, 10]. Either crossed or uncrossed fusion is appropriate. The entire fused image will shimmer.

A second perception was recognized by Dove [8, 9, 10] and is called stereoscopic or binocular luster. Under similar circumstances to those that cause the Venetian blind effect, i.e., when there is an adequate luminance disparity while viewing a single stimulus, the image appears to have a luster like the shimmer on a body of water or reflective piece of metal (Fig 2).

**Fig 3:**
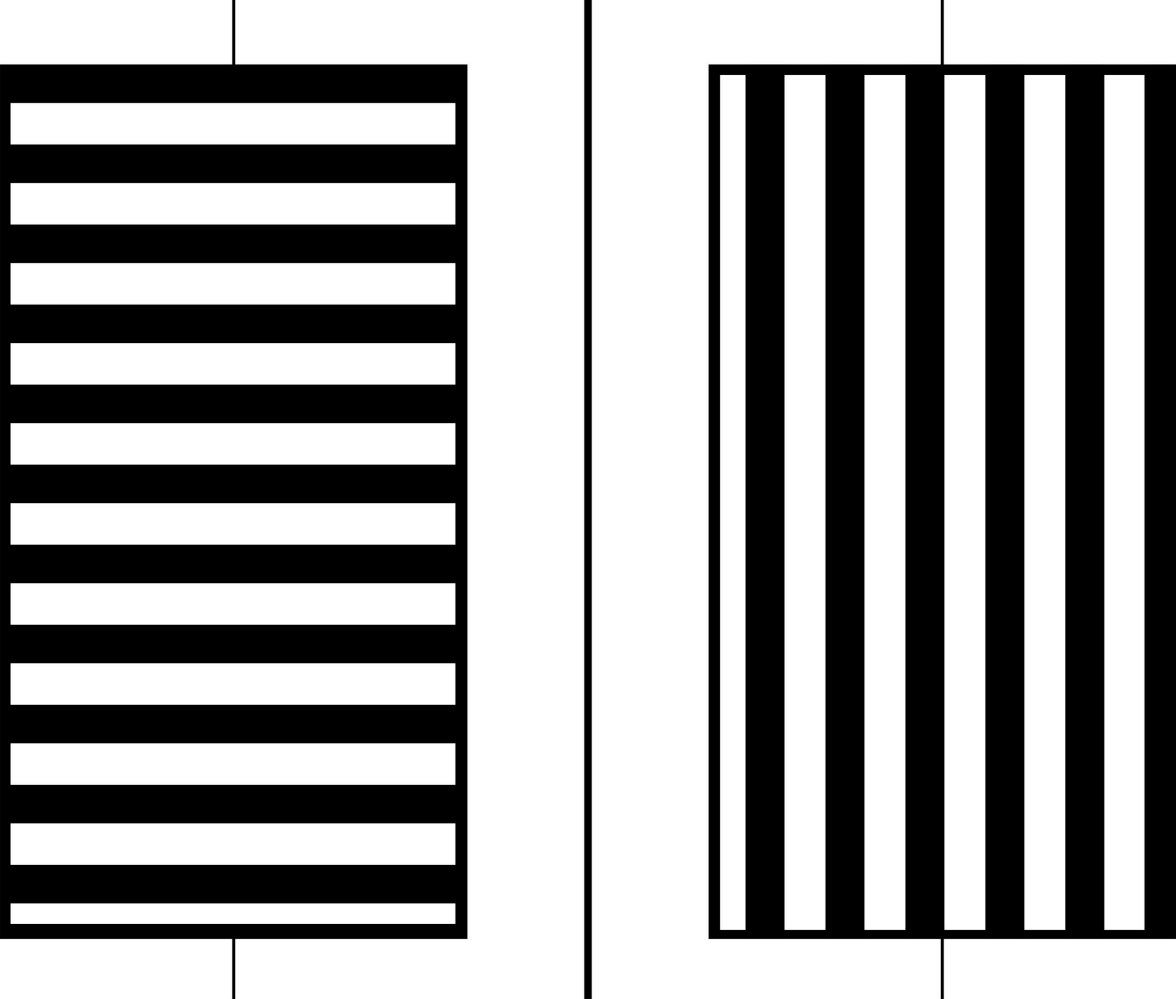
Binocular rivalry example. Stereogram of gratings image with a geometric disparity for demonstrating binocular rivalry, taken after Panum [15]; Panum's gratings were at diagonals instead of horizontal and vertical). Either crossed or uncrossed fusion is appropriate. The entire fused image will rival.

Finally, there is binocular rivalry [2]. Many researchers (see, e.g., [11, 12]) have studied binocular rivalry, where it becomes nearly impossible to maintain a fused binocular image as perception wavers stochastically back and forth between the two eyes' views (Fig 3). Though generally discussed in terms of geometric disparity and, occasionally, disparity in color or other qualities of vision, binocular rivalry has also been studied using geometrically identical images that are presented either with luminance disparities [13] or without [14].

These three percepts have not systematically been explored in relation to one another. Our informal observations suggest that binocular luster can occur with contrast disparities. It may not be meaningful to discuss binocular rivalry with a contrast disparity since, at high contrast disparities, one monocular image with very low contrast may be wholly suppressed while the other image dominates perception without rivalrous alternation. Some researchers state that luster and rivalry co-occur (e.g., [16]) while others state that luster appears only when there is no rivalry (e.g., [9], pp. 493–528; [10], pp. 197–316). Finally, the relationship between luster and rivalry may depend on other factors such as the size of the images (e.g., [13]).

It is clear that the visual system responds to luminance and contrast information with multiple perceptions, including brightness and perceived contrast (see, e.g., [7, 17]). Our goal is to clarify the behavior of the visual mechanisms involved. If two perceptual phenomena fail to arise in similar circumstances and fail to co-vary, then it is reasonable to presume that they arise from distinct underlying mechanisms. As well, by the simplest of psychophysical linking hypotheses ([18], p. 144), "… if two sensations are discriminable then their underlying physiological states must also differ" ([19], p. 1236). As all of the perceptual phenomena listed above arise from similar stimulus manipulations, any distinction would be informative for understanding the mechanisms in processing binocular luminance and contrast information. Of course, given that we are considering three distinct perceptual qualities, the physiological states underlying each must be distinct. Our question concerns the degree to which the stimulus configurations that engender each of these qualities overlap and/or co-vary as an indicator of distinct underlying mechanisms.

We demonstrate that the Venetian blind effect, binocular luster, and binocular rivalry can be perceived in the same square-wave grating by manipulating luminance or contrast disparity (though no binocular rivalry occurred with just a contrast disparity). The key stimulus feature for perceiving luster and rivalry is the relation between each monocular grating image and the background luminance. Given a minimum amount of luminance or contrast disparity, a perception of luster occurs when luminance values of the monocular images "straddle" the background luminance (one eye’s image has a higher luminance than the background and the other eye’s image has a lower luminance than the background). Rivalry follows as the luminance disparity increases. Perceived rotation, or the Venetian blind effect, can be described with reference to the average luminance and contrast disparity of the viewed gratings, ignoring whether or not the monocular images straddle the background luminance. We argue that the three visual phenomena must be controlled by at least two underlying mechanisms for processing binocular luminance and contrast information: one supporting the perception of rotation and the other that of luster and/or rivalry. Again, the latter mechanism must involve more than one physiological state as the perceptual qualities of luster and rivalry differ. Our results cannot be explained by other processing mechanisms, e.g., the processing of geometric disparity that is also known to induce binocular rivalry.

### 1.1 Perceived rotation

The Venetian blind effect, or perceived rotation engendered by a luminance disparity or contrast disparity, was modeled by Cibis and Haber [5] as a geometric disparity created by a particular form of irradiation (see [20], pp. 186–193). Assuming that the perceived edge of a light bar at 100% contrast corresponds to the place where retinal illuminance crosses threshold, they theorized that the perceived edge of a bar would vary with retinal illumination since the optics of the eye smear the retinal image of the bar. Hence, when a square-wave grating is viewed with either a luminance or a contrast disparity, the light bars of the more intense retinal image will appear wider than those in the corresponding less intense image on the other retina, giving the hypothesized perceived rotation based on a perceived geometric disparity.

A separate irradiation model was developed by Filley et al. [6] based directly on Helmholtz’s description of irradiation ([20], pp. 186–193). Helmholtz noted that the optical smearing of the retinal image, coupled with the compressive non-linearity of the response of the retina to illumination, implies that the perceived edge between a light and dark region will be shifted away from the light region regardless of just how the visual system defines the location of that edge. Filley et al. [6] developed a mathematical model of this form of irradiation using the point-spread function of the eye (cf., [21]) coupled with the Naka-Rushton compressive non-linearity [22].

The Cibis-Haber model predicts that the Venetian blind effect will not be visible at moderate contrasts since the region between the light bars of a given square-wave grating will be above retinal illuminance threshold. Both the Cibis-Haber and the von Helmholtz-inspired irradiation models predict that perceived rotation will vary with edge blur width since the apparent location of a blurred edge varies with the blur width (e.g., with sine-wave blur: [23]; with Gaussian blur: [24, 25]). Finally, since both models ascribe the Venetian blind effect to geometric disparities, both predict that the Venetian blind effect will have the same temporal dynamics as geometry-based stereopsis. All three of these predictions fail [6, 26].

The intensity difference model, initially developed by Filley et al. [6], successfully describes the probability of seeing rotation as a function of luminance and contrast disparities. Hetley and Stine [7] extended this difference model to the magnitude of perceived rotation in the Venetian blind effect. For the effect of contrast disparity, the model compares the output of two Naka-Rushton equations [22] emulating the response of two different neurons, one responding to the left monocular image and the other to the right. The effects of luminance are modeled through the Naka-Rushton equation by varying the maximum response rate of the cell (R_max_; see Experiment II). Dobias and Stine [26] further generalized this model to account for the effects of very large luminance or contrast disparities, as discussed in Experiment II. The two major conclusions from this work are that perceived rotation in the Venetian blind effect can be described using the inter-ocular difference in luminance or contrast of the stimulus and that perceived rotation due to a luminance or contrast disparity is the result of a physiological response rather than just irradiation.

### 1.2 Binocular luster

Much of the analysis of binocular luster has been qualitative. Many writers (e.g., [27], chapter 15; [28], section 52; [16, 29]) merely mention luster. It has been described as the sheen of a crystal or metal surface, a natural result of specular reflection causing a binocular luminance disparity (see [29] for diagrams; [30]). Many demonstrations use maximum luminance disparity, i.e., completely black components in one monocular image and white components in the other, to induce a perception of luster (Fig 2, as described in [9, 10]).

Wolfe and Franzel [31] noted that luster seems most compelling when one monocular image is more luminant than the background and the other is less luminant, thus dichoptically straddling the background. Anstis [32] later demonstrated that dichoptic straddling is the ideal condition for inducing a perception of luster, regardless of the specific value of the background luminance. Luster was still perceived when the squares were very close to the background without straddling it; but unlike with the straddling case, luster decreased with increasing distance in luminance, and the decrease was symmetrical with distance above versus below the background luminance. Generally, larger contrasts are required for binocular luster when dichoptically-presented images have a common contrast polarity than when they are of opposite polarity [33, 34]. Further, dichoptic luminance discrimination thresholds tend to be smaller than those to report binocular luster [35]. At threshold, dichoptic color differences can appear lustrous [36] (see also [37]).

Georgeson, Wallis, Meese, and Baker [38] found that a luster response had to be included into a model of binocular contrast discrimination in order to describe results with dichoptically-presented, horizontally-oriented pair of sine-wave gratings that were *π* radians out of phase (so, each point in the left eye’s grating had the opposite contrast polarity to the corresponding point in the right eye’s grating relative to the background). With added noise, their model also described data from Anstis [32]. For dynamic stimuli, where a pair of spatially-identical dichoptic stimuli are presented briefly with a temporal phase difference, ratings of luster seem to be maximized when the contrast of the two stimuli have opposite-sign slopes in time (i.e., one member of the pair has increasing contrast while the other has decreasing contrast) and they are presented on a relatively dark background [39]. Straddling the background did not enhance luster in this case.

Writers have debated binocular luster's relationship to binocular rivalry. Helmholtz [9, 10], Ludwig, Pieper, and Lachnit [40], and Tyler [41] argued that luster did not depend on the shifts in perception over time that occur in rivalry. Rather, luster is a result of a stable perception of a fused image. However, Dove (as described in [9], p. 514) reported seeing luster in rivaling images during the precise moments where perception was shifting from one monocular image to the other. Further, Julesz and Tyler [16] observed luster when images rivaled but not when images were fused. Birnkrant, Wolfe, Kunar, and Sng [42] described luster as "dynamic," like rivalry. There are therefore at least two views on the qualitative nature of binocular luster: as a phenomenon tied to binocular rivalry, and as a phenomenon on its own.

On the relationship between binocular luster and depth perception, McCamy [29] and Tyler [41] stated that binocular luster involves some indeterminate impression of depth. Tyler's description suggested that, although research participants can use luster to inform them when a stereographic image has a binocular disparity, luster alone has little use in judging what depth is simulated in the image. Mausfeld, Wendt, and Golz [39] suggest that a small perceived depth separation between luster and the viewed surface is indicative of a separation of accidental features, due to the illuminant, and essential features of the surface. Howard [13] tied the perception of depth, luster, and rivalry together as discussed in the section on binocular rivalry, below.

Lastly, binocular luster in combination with several monocular cues is important to the perception of a surface material's "glossiness," as opposed to "roughness" or other quality [30, 39, 43–48]

### 1.3 Binocular rivalry

Binocular rivalry, recognized for nearly 2000 years [49], has been studied extensively (see [11, 12, 50, 51]). Wheatstone [2] reported that two images often fragment during rivalry, with the viewer perceiving a fractured mosaic between periods where a single image dominates. Various sources (e.g., [40, 52]; [53], p. 327; [54]) have described how the mosaic or piecemeal dominance during transitions only occurs for larger images. For images 1° in visual angle or smaller, exclusive or unitary rivalry may occur where perception changes as a whole.

Howard [13] (see also [54]) found that circles smaller than 1°, with one monocular image black and the other white, result in unitary rivalry with a perception of being more distant than their surroundings, which he called the sieve effect. Circles larger than 1° result in mosaic dominance, binocular luster, and an indeterminate depth. Howard proposed that the perception of luster occurs in this situation because binocular brightness summation is possible during mosaic dominance. Matsumiya, Howard, and Kaneko [55] later supported the correlation between the perceived depth of the sieve effect and the incidence of unitary rivalry.

However, Hetley and Stine's [7] participants informally noted luster with square-wave gratings that were less than 1° in size, and without rivalry. It is possible that the use of gratings gives results that differ from the use of Howard's [13] solid circles.

There is some indication that the circumstances that give rise to luminance-based rivalry may overlap with those that cause binocular luster. Fry and Bartley [56] informally noted that luminance values dichoptically straddling the background led to binocular rivalry. This is the same condition shown by Anstis [32] to be ideal for inducing luster.

### 1.4 Current research

We performed two experiments to quantify the relationships among perceived rotation through the Venetian blind effect, binocular luster, and binocular rivalry, as based on binocular luminance and contrast information. If two perceptual phenomena fail to arise in similar circumstances or to co-vary, then it is reasonable to presume they arise from distinct underlying mechanisms, and it has been seen before [7] that luminance and contrast information may be used differentially for different perceptions. In Experiment I we measure the incidence of perceived rotation, luster, and rivalry as a function of average luminance and contrast disparity using square-wave gratings. We then measure perceived rotation, luster, and rivalry in Experiment II using isolated light bars and dark bars taken from the square-wave gratings of Experiment I. Decomposing the stimuli from Experiment I enables us to test the generalized difference model of perceived rotation, the role of straddling the background for perceiving luster and rivalry simultaneously, and the degree to which rotation is perceived with luster and/or rivalry.

## 2. Material and methods

### 2.1 General methods

We performed two experiments with the same participants and apparatus, except as noted. In all experiments, we used the method of constant stimuli to determine the circumstances under which the participants perceived the three phenomena of interest: perceived rotation (the Venetian blind effect), binocular luster, and binocular rivalry.

#### 2.1.1 Participants

All participants were adult males and have had experience with stereoscopic viewing. Participants WWS and JJD had normal vision, while participant RSH had myopia as well as an astigmatism in the left eye, which were corrected by glasses. University of New Hampshire Institutional Review Board clearance was acquired beforehand and all participants gave informed consent.

#### 2.1.2 Apparatus

All experimental sessions were performed in a darkened room. One participant at a time was seated, bit onto a bite bar, and viewed stimuli through 3 mm artificial pupils in order to control for pupil-size fluctuations (see, for example, [57, 58]). The experiment was controlled by a program running in Mathematica 4.0.2.1 on a Power Mac G4, displayed on an Apple ColorSync Display. Vertical baffles were in place along the participant's line of sight to separate the views for the two eyes. The display was 1.62 m in front of the participant giving a single pixel width of around 46.2 seconds of visual angle. The entire viewing area was 3.8° in width (7.7° in total, separated for the two eyes and with a small amount covered by the baffles) and 4.6° in height, surrounded by a cardboard mask. Each monocular image was centered in the left or right half of the screen with a vertical dark line above and below (to aid in fusing) and with other characteristics that varied based on the experiment. All experimental images were on a background of uniform gray (42.5 cd/m^2^, or 300 photopic td).

#### 2.1.3 Procedure

The participant bit onto the bite bar and aligned each artificial pupil with the assistance of the experimenter or a trained participant. The experimenter/assistant darkened the room and exited, and the experimental session began when the participant entered a key on a keypad. The sample stimulus was replaced with a uniform gray (at the background luminance) for a five-minute adaptation period. Experimental trials began afterwards. The participant was shown a binocular image for 5 seconds, which was chosen pseudorandomly from the available conditions for that experiment. The stimulus was then replaced with the uniform gray again and the participant was prompted to respond. After the response was entered on the keypad, the uniform gray remained on the screen for an interstimulus interval of 5 seconds, and then the next trial began.

Stimuli are described in the sections for each experiment, below, and in S1–S6 Tables. Images were defined in terms of average luminance, contrast, and the disparity in those same values between the views of the two eyes. Contrast was calculated as Michelson contrast ([59], p. 40).

The participant's task was to make three judgments for each binocular image, reporting whether the image appeared to have a rotation in depth (the Venetian blind effect), binocular luster, and/or binocular rivalry. For perceived rotation, direction of rotation was not measured. For binocular luster, participants were instructed to respond to a "glow," regardless whether it was perceived as stable luster (e.g., [9, 10]) or as transient luster tied to alternations in rivalry (e.g., Dove, as described [9], p. 514). For binocular rivalry, participants were instructed to respond to either unitary or mosaic rivalry (see, e.g., [53], p. 327).

Participants performed practice sessions until they felt comfortable and responses stabilized. For Experiments I and II, these were: for JJD, 2 and 2 sessions; for RSH, 1 and 3 sessions; for WWS, 2 and 2 sessions, respectively. They then performed formal sessions until 12 trials were completed for every condition in that experiment. Because of varying numbers of conditions, this meant 3 total sessions in Experiment I and 6 in Experiment II.

#### 2.1.4 Data analysis

**Fig 4:**
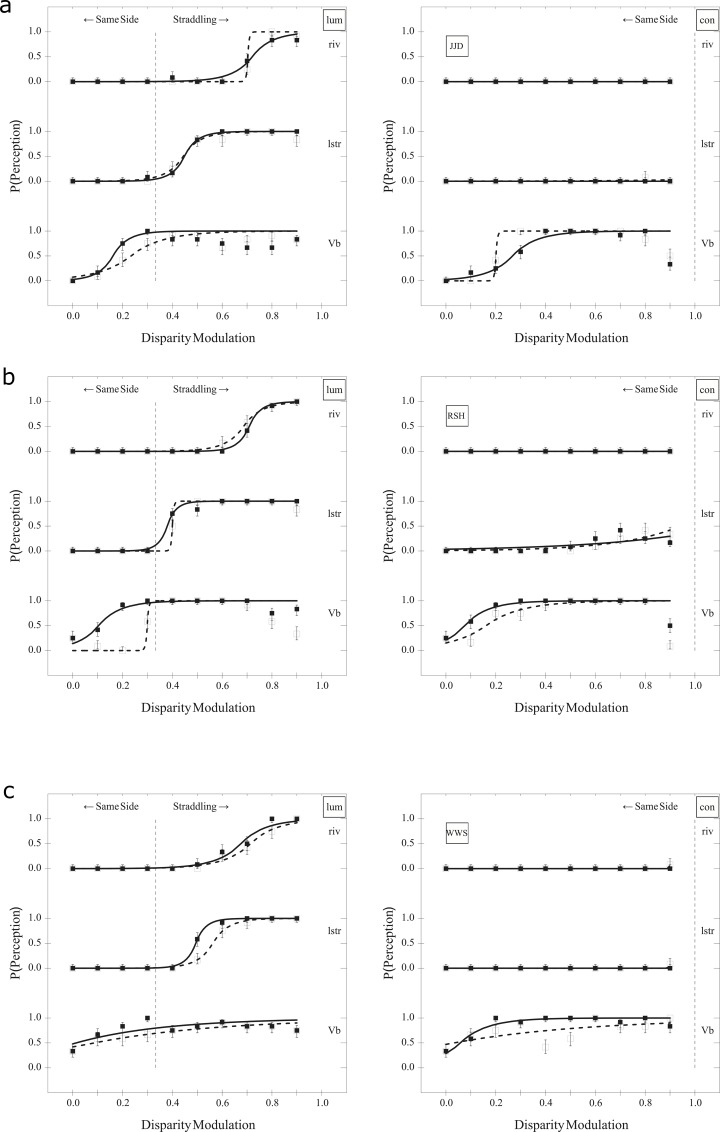
Experiment I results. Data for (a) participant JJD, (b) RSH, and (c) WWS. Each plot, from top to bottom, shows probability of detecting rivalry ("riv"), luster ("lstr"), and rotation ("Vb") at different modulations. The left plots are for luminance ("lum") and right are for contrast ("con"). Filled boxes are for the left "eye" condition and empty boxes for right "eye." The vertical dotted line is the point where the light bars begin to straddle the background. Error bars indicate one standard error based on the score estimator [60] (see also [61, 62]). Open squares represent the probability of responding "present" for perceived rivalry (riv), luster (lstr), and rotation (the Venetian blind effect, Vb) when the right eye viewed the stimulus with higher average luminance or contrast and filled squares represent the case when the left eye viewed the stimulus with higher average luminance or contrast. Curves are least-squares fits of Laplace cumulative probability distributions, with dashed for the case when the right eye viewed the stimulus with higher average luminance or contrast and solid for the case when the left eye viewed the stimulus with higher average luminance or contrast [6].

The data were plotted as the probability of responding "present" to each perceptual phenomenon across the 12 trials for each condition, using standard error bars based on the score estimator [60] (see also [61, 62]). Thresholds for the perception of each phenomenon were calculated by fitting curves to the data. These curves were the cumulative density function of a Laplace distribution [6], fit using the FindFit function in Mathematica 5.0.0.0. When there was no fit found to the data, the results of this function were not plotted. Note that in some plots (e.g., Fig 4) some of the fits appear more sharp or steplike than necessary to fit the data. These fits were checked by varying the starting values for the FindFit function.

### 2.2 Experiment I: Three perceptual phenomena using a grating stimulus

#### 2.2.1 Rationale and predictions

We performed Experiment I to measure the range of average luminance and contrast disparities over which perceived rotation, luster, and rivalry would be reported. Rotation should be reported over a wide range of disparities with lower and upper bounds [6, 7] and [26], given the existence of threshold disparities for perceiving rotation as well as disparities so large that one eye's stimulus dominates perception.

The onset of luster should be associated with that disparity where the luminance of the stimulus bars either straddles the luminance of the background [31] or, perhaps, is close to straddling the background [32, 38]. At large luminance disparities, we would expect to see rivalry with luster [13]. As mentioned, our informal observations suggest that the onset of luster, which should be associated with straddling the background, will occur before the onset of rivalry. We did not expect rivalry with a contrast manipulation, though the perception of rotation and luster should occur as described.

#### 2.2.2 Stimuli

Experiment I used square-wave gratings made up of three light bars and four dark bars at a spatial frequency of 1.5 cycles per degree (akin to Fig 1). Each monocular image was around 2.3° in width and 1.5° in height. The images varied from a monoptic "neutral" state, with no dichoptic luminance or contrast modulation,^2^ where the light and dark bars averaged 42.5 cd/m^2^ (the base luminance) and had a contrast of 0.5 (the base contrast). (We measure disparity magnitude with dichoptic luminance modulation and dichoptic contrast modulation, after [7]. Dichoptic luminance modulation is defined by the equation

$$DichopticLumMod=\frac{L_{l}-L_{r}}{L_{l}+L_{r}},$$

where L_eye_ is the average luminance of the grating presented to an eye denoted by subscript, (l)eft or (r)ight. L_eye_ can be calculated using

$$L_{l}=\frac{L_{l}+L_{r}}{2}(1+DichopticLumMod)$$

and the equivalent for L_r_, subtracting modulation instead of adding. We abbreviate this as

$$L_{eye}=(BaseLuminance)*(1\pm DichopticLumMod),$$

where BaseLuminance is the mean of the average luminance values of the two monocular images. One monocular image therefore averages above the base luminance and the other averages below, with a magnitude determined by the modulation. The same general equation holds for dichoptic contrast modulation, in Michelson contrast ([59], p. 40), describing monocular contrasts relative to a base contrast.) Some images had dichoptic luminance modulation and some had dichoptic contrast modulation. The remaining area on the screen was at the background luminance of 42.5 cd/m^2^.

There were three independent variables: whether dichoptic luminance modulation or dichoptic contrast modulation were presented, the amount of the modulation, and whether the left or right eye received the image with higher luminance or contrast. Possible modulation values for either luminance or contrast varied in 0.10 increments from 0.10 to 0.90 (see S1 and S2 Tables), with an extra neutral condition that had no modulation. Each combination of values, including the neutral condition, appeared four times in one session. Participants performed three sessions, therefore completing 12 trials for each condition.

#### 2.2.3 Results

Data are shown in Fig 4, plotting the probability of responding "present" for perceived rivalry (riv), luster (lstr), and rotation (the Venetian blind effect, Vb), with one pair of subplots for each participant. Probabilities as a function of luminance disparities are shown in the left panel and contrast disparities in the right. The vertical dotted lines indicate the disparity at which one of the gratings' bright bars fall below the luminance of the background. Hence, at disparities beyond the vertical dotted lines, the luminance of the bright bars of the dichoptic pair of gratings straddle the background luminance. At disparities below the vertical dotted lines, the luminance of all of the visible bright bars is above the background luminance.

For all three subjects, thresholds (modulation engendering a 0.5 probability of response) for rotation were below those for luster, which, in turn, were below those for rivalry when viewing luminance modulations. Neither luster nor rivalry was seen with contrast modulations, though rotation was perceived. Finally, for two of three subjects, a drop in rotation was perceived at modulations approaching 0.9.

JJD may have exhibited some evidence of a stronger input from the left eye for perceived rotation with a luminance disparity and for the right with a contrast disparity. RSH showed a stronger input from the left eye for luminance disparities and, perhaps, for contrast disparities. WWS showed, perhaps, a stronger input for perceived rotation from the left eye for both luminance and contrast disparities.

Participants reported informally that all rivalry with these images was mosaic. Further, sustained luster was reported for lower to moderate luminance disparities while transient luster was reported for higher disparities.

#### 2.2.4 Discussion

All three subjects exhibited different thresholds for perceived rotation, luster, and rivalry with luminance disparities, demonstrating differing onsets for the three perceptions and that all three may be seen simultaneously. However, only perceived rotation was evident with contrast disparities, suggesting that the mechanisms underlying perceived rotation, or the Venetian blind effect, are distinct from those underlying luster and rivalry.

Interocular differences in the strength of input to perceived rotation replicated that reported for RSH and WWS by Hetley and Stine [7]. Finally, with the informal reports, mosaic rivalry is to be expected given the images are larger than 1° in visual angle (see, e.g., [53], p. 327). Replicating Dove's (as described in [9], p. 514) observation, transient luster was perceived only at higher luminance disparities, where rivalry also occurs.

Our results suggest that the luminance of the bright bars must straddle the background luminance before luster and rivalry reach threshold. Further, only the light bars control this threshold. Anstis [32], Fry and Bartley [56], and Wolfe and Franzel [31] mention the need for images dichoptically straddling the background in order to see luster and/or rivalry, though clearly luster is possible without straddling the background [33, 34, 38, 39].

### 2.3 Experiment II: Light bars and dark bars in isolation

#### 2.3.1 Rationale and predictions

We performed Experiment II to measure the effects of individual bar luminance on perceived rotation, luster, and rivalry while holding the luminance surrounding the bars constant. The bar luminance values were taken from Experiment I stimuli. Specifically, our stimuli used bright bars with luminance that matched either (1) the average luminance of the monoptic stimuli from Experiment I with average luminance disparities, (2) the luminance of the bright bars of the monoptic stimuli from Experiment I with average luminance disparities, (3) the luminance of the bright bars of the monoptic stimuli from Experiment I with contrast disparities, (4) the luminance of the dark bars of the monoptic stimuli from Experiment I with contrast and average luminance disparities (dark bars covered the same luminance range regardless of which type of disparity was used). We call the independent variable composed of these four conditions the *luminance source*, which is described further in the section on stimuli, below.

We manipulated the luminance of the bars to vary exactly as they had varied in Experiment I, creating what we term "Experiment I equivalent disparity modulation." When coupled with the constant background luminance, both contrast and average luminance vary in each monoptic image with Experiment I equivalent disparity modulation (at times non-monotonically). This manipulation allowed us simultaneously to test a prediction from Hetley and Stine [7] concerning the relationship of contrast and average luminance disparity to perceived rotation, to test whether or not it is critical for the perception of luster and rivalry that the luminance of the bright bars of a dichoptic pair of gratings straddle the background luminance, and to measure the degree to which perceived rotation, luster, and rivalry co-vary across these stimulus manipulations.

That both contrast and average luminance disparities engender perceived rotation is now well established ([2, 3, 4, 5, 6] and [26]). A generalized difference model describes perceived rotation as a function of contrast disparities that range from threshold to disparities so large that one image dominates perception (Appendix A in [26]). Hetley and Stine [7] modeled perceived rotation as a function of contrast and average luminance disparities assuming that neural responses to contrast and average luminance may be described using the Naka-Rushton equation [22] with parameters measured by Sclar, Maunsell, and Lennie [63] from neurons in macaque monkey (Macaca fascicularis) striate cortex in response to contrast and by Geisler, Albrecht, and Crane [64] from neurons in cat (Felis catus) striate cortex in response to average luminance. The resulting model described the response of a cortical neuron to contrast and average luminance as

$$R(C,L)=\frac{R_{max}(L)C^{n}}{C^{n}+\sigma_{50}^{n}}+M$$ (1)

where

$$R_{max}(L)=\frac{R_{{max}_{L}}L^{n_{L}}}{L^{n_{L}}+\sigma_{50}^{n_{L}}}+M_{L}.$$ (2)

R(C, L) is the response of a neuron to a grating of contrast C and average luminance L, and R_max_(L) is the maximum possible response to a grating with average luminance L. For Eq (1), M is the spontaneous rate of response, σ_50_ is the contrast that causes half of the maximum response, and n is a parameter that adjusts the steepness of the response, with R_maxL_, n_L_, and σ_50_, sharing analogous definitions in Eq (2). Effectively, Eq (1) states that a cell in V1 responds to contrast following the Naka-Rushton equation with the maximum response rate modulated by the average luminance of the stimulus.

Generalizing the work of Hetley and Stine [7], Dobias and Stine [26] modeled the perceived shift in the edge of each bright bar of a square-wave grating as a function of the contrast disparity of the grating. Adapting the model for the manipulation of contrast and average luminance disparities through Eq (1), the generalized intensity difference model describes the shift in bar edge as

$$EdgeShift(C_{l},L_{l},C_{r},L_{r})=gain\left(R(C_{l},L_{l})-R(C_{r},L_{r})\right)(R(C_{l},L_{l})-M_{ES})(R(C_{r},L_{r})-M_{ES})$$ (3)

with a resulting perceived horizontal size ratio^3^ of

$$PercHSR(C_{l},L_{l},C_{r},L_{r})=\frac{width+2EdgeShift(C_{l},L_{l},C_{r},L_{r})}{width-2EdgeShift(C_{l},L_{l},C_{r},L_{r})},$$ (4)

where width is the width of a single bright bar of the grating, C_eye_ and L_eye_ are the contrast and average luminance of the square-wave grating viewed by eye, gain is a multiplicative gain constant, and M_ES_ is a constant that describes when a disparity becomes so large one eye’s image suppresses the other eye’s image, effectively giving an edge shift of zero. (We measure geometric disparity either as visual angle or as horizontal size ratio (HSR). In Backus, Banks, van Ee, and Crowell [65], the HSR is the ratio of the visual angle of the left monocular image and the right monocular image.) Perceived rotation is directly related to perceived horizontal size ratio ([65], Equation (A5); which is repeated in [26], Equation (B1)).

The generalized intensity difference model should describe variations in the probability of perceiving rotation as a function of Experiment I equivalent disparity with a single set of parameters across all conditions. However, only those instances where the luminance of the bright bars straddle the background, or nearly straddle the background, should create the perception of luster, with rivalry appearing as the luminance disparity increases.

#### 2.3.2 Stimuli

**Fig 5:**
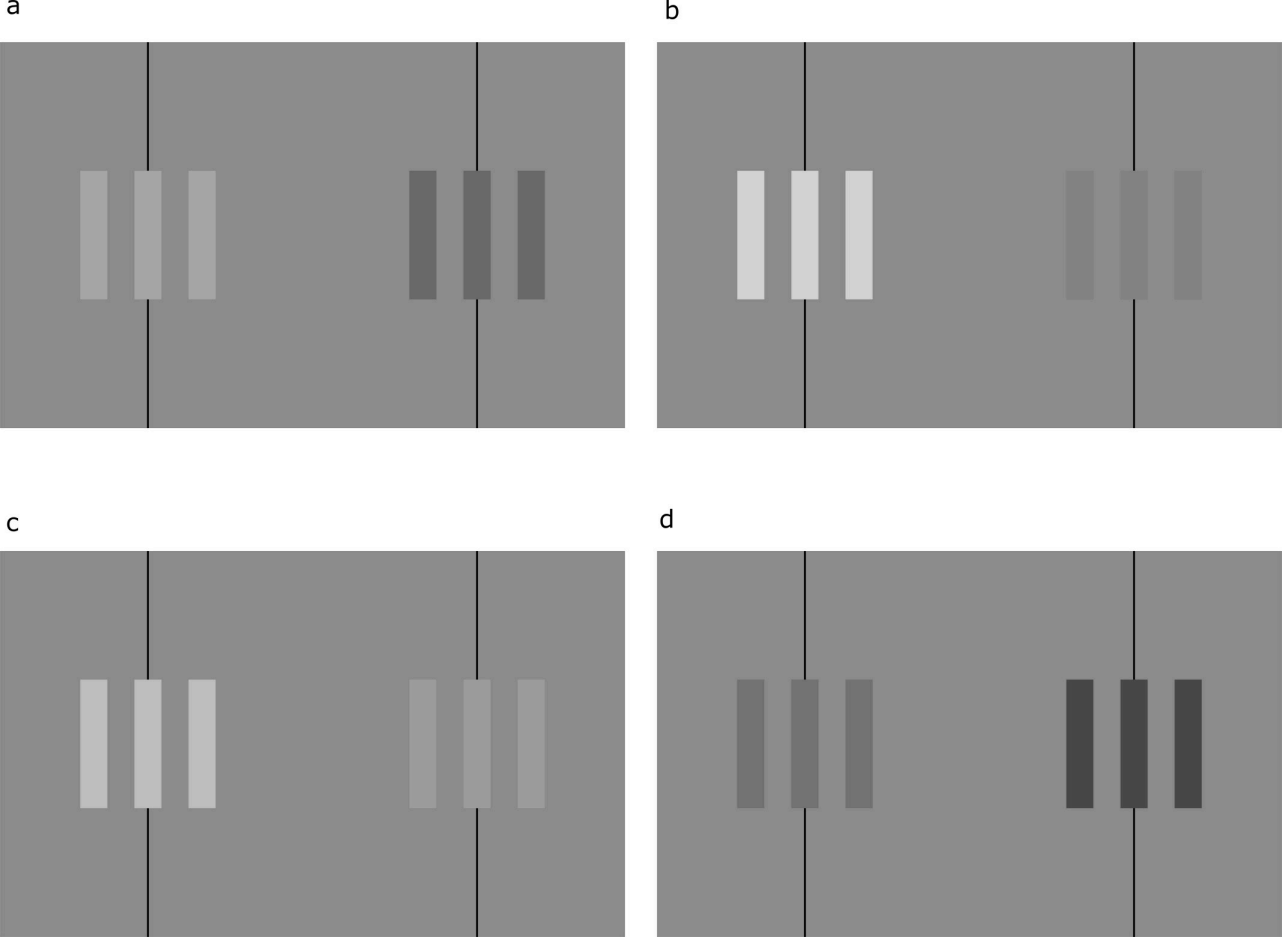
Sample Experiment II stimuli. (a) The "average luminance" condition, with luminance values derived from a grating with dichoptic luminance modulation of 0.4. (b) "Light luminance bars," from dichoptic luminance modulation of 0.4. (c) "Light contrast bars," from dichoptic contrast modulation of 0.5. (d) "Dark bars," from dichoptic luminance modulation of 0.4. The bars straddle the background in (a) and (b), and not in (c) and (d).

Experiment II used images containing three dichoptic bars on a 42.5 cd/m^2^ uniform field (Fig 5). The three bars were the same dimensions and position as the three light bars in the grating stimulus in Experiment I, each being 0.3° in width and 1.5° in height, separated by one bar width from each other. The background gray continued between the bars.

The bars varied in one of four ways (as described in the section on rationale and predictions and listed in S3–S6 Tables). In the "average luminance" condition, the bars were at the average luminance values of the monoptic stimuli used in Experiment I (Fig 5A).

In the "light luminance bars" (Fig 5B) and "light contrast bars" (Fig 5C) conditions, the bars were at the luminance values of the light bars of a square-wave grating that had the dichoptic luminance modulations or dichoptic contrast modulations from Experiment I, respectively. For both of these conditions, modulation of the grating from which the plain bars were taken varied in 0.10 increments from 0.10 to 0.90.

In the "dark bars" condition (Fig 5D), the images were at the luminance values of the dark bars in a square-wave grating. In Experiment I, the spread of dark bar luminance values during the luminance modulation coincided with that during the contrast modulation, and only swapped whether the left or right image had the highest luminance; so there was only one "dark bars" condition to represent both luminance and contrast disparities. The modulation values again varied in 0.10 increments from 0.10 to 0.90. Though there were four dark bars in each original grating, only three were presented here in order to make the stimuli more comparable across conditions.

The average luminance of gratings with a luminance disparity was always centered on the base of 42.5 cd/m^2^, and so the "average luminance" plain bars always had that base luminance. The light bars of gratings with either a luminance or contrast disparity always averaged 63.75 cd/m^2^, and so the "light luminance bars" and "light contrast bars" images always had that base luminance. The dark bars of gratings with either a luminance or contrast disparity always averaged 21.25 cd/m^2^, and so the "dark bars" images always had that base luminance.

The "average luminance," "light luminance bars," and "dark bars" images can still be said to have dichoptic luminance modulation from 0.10 to 0.90. However, for the "light contrast bars" condition, the recalculated dichoptic luminance modulation proceeds from 0.03 to 0.30 in increments of 0.03.

In total, there were three independent variables: the "luminance source," the modulation amplitude, and whether the left or right eye received the image with higher luminance, which were factorially combined. There were also three neutral conditions: one for "average luminance," one for both "light luminance bars" and "light contrast bars" (as these are identical with no modulation), and one for "dark bars." Note that the neutral condition for "average luminance" is a screen that is blank gray except for alignment lines, and so even though this condition was presented, it will not be plotted in this experiment. Each combination of values, including the neutral conditions, appeared twice in one session. Participants performed six sessions, therefore completing 12 trials for each condition.

#### 2.3.3 Results

**Fig 6:**
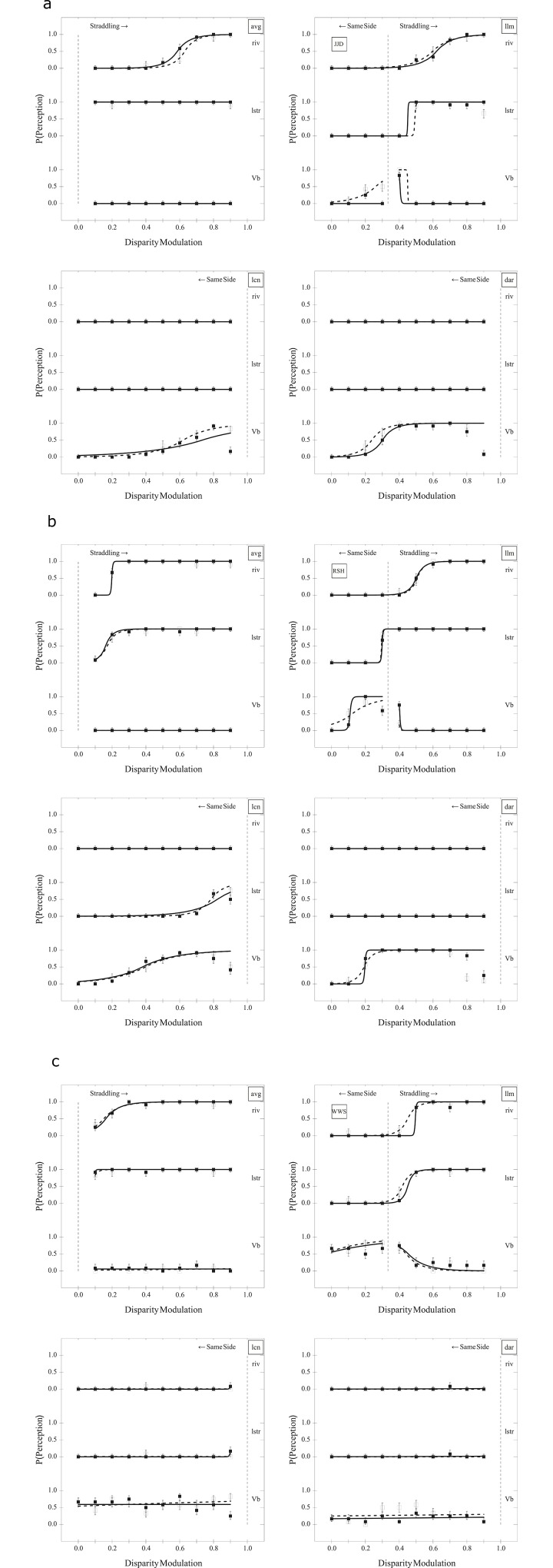
Experiment II results. Data for participants (a) JJD, (b) RSH, and (c) WWS. From top to bottom for each plot, probability of detecting rivalry ("riv"), luster ("lstr"), and rotation ("Vb") at different modulations. The top left plot is for "average luminance" ("avg"), top right "light luminance bars" ("llm"), bottom left "light contrast bars" ("lcn"), and bottom right "dark bars" ("dar"). Filled boxes are for the left "eye" condition and empty boxes for right "eye." The vertical dotted line is the point where the light bars begin to straddle the background. Error bars indicate one standard error based on the score estimator [60] (see also [61, 62]). Open squares represent the probability of detecting rivalry ("riv"), luster ("lstr"), and rotation ("Vb") when the right eye viewed the bars with the higher luminance and filled squares represent the case when the left eye viewed the bars with the higher luminance. Curves are least-squares fit of Laplace cumulative probability distributions, with dashed for the case when the right eye viewed the bars with the higher luminance and solid for the case when the left eye viewed the stimulus with higher luminance [6].

Data are shown in Fig 6, again plotting the probability of responding "present" for each percept as a function of modulation amplitude. Note that the modulation values at the bottom of each plot are the dichoptic luminance or contrast modulation values in the original grating images of Experiment I, allowing direct comparison of these plots to those in Fig 4. That is, the "average luminance" (avg) and "light luminance bars" (llm) plots can be compared to the plots for images with dichoptic luminance modulation, the "light contrast bars" (lcn) plots can be compared to the plots for images with dichoptic contrast modulation, and the "dark bars" (dar) plots can be compared to both luminance and contrast modulations. Again, the vertical dotted lines indicate the disparity at which one of the dichoptic gratings pairs' bright bars fall below the luminance of the background.

The "light luminance bars" and "light contrast bars" plots exhibit well-defined thresholds for the initial perception of binocular rivalry (riv) and binocular luster (lstr) that mirror those in Experiment I. The threshold for rivalry was above that for luster, both could be seen at one time, and, with the possible exception of RSH seeing luster (Fig 6B), both were seen only when the luminance of the bars straddled that of the background. RSH saw, perhaps, some luster at a modulation just below that where the luminance of the bars straddles the background in the light luminance bars and light contrast bars conditions, though he also had a clearly measurable threshold disparity for luster in the average luminance condition.

JJD and RSH showed clear threshold disparities for rotation, or the Venetian blind effect (Vb), in the light bar contrast and dark bar conditions (Fig 6A and 6B). Further, both subjects showed a drop in the probability of seeing rotation with very large disparities. WWS showed a low and relatively constant probability of seeing rotation during the light bar contrast condition and a very low probability in the dark bar condition (Fig 6C). In the light luminance bars condition all three subjects perceived rotation when the luminance disparity was of an amplitude that just straddled the background, with little or no rotation at higher amplitudes. At lower amplitudes JJD and RSH showed a drop in the probability to see rotation while WWS showed, again, a low and relatively constant probability.

Our subjects showed little effect of the eye viewing the more-intense stimulus. Participants again noted, informally, that these images only underwent mosaic rivalry (see, e.g., [53], p. 327).

#### 2.3.4 Modeling perceived rotation

The probability of perceiving rotation, or the Venetian blind effect, showed both lower and upper threshold luminance disparities. In order to generate specific descriptions from the generalized difference model, we fixed the values for all of the constants to those used in Hetley and Stine ([7]; R_maxL_ = 1, σ_50_ = 0.15, n = 2.4, n_L_ = 3.0, M = 8.22, M_L_ = 0.0, and gain = 1.0) with the exception of M_ES_. Perceived horizontal size ratios were then calculated for each Experiment I equivalent disparity presented across the four conditions. The Mathematica 11 Interpolation module was used to build a spline interpolation to generate a continuous predicted perceived horizontal size ratio function from the model. Using a hierarchical approach, that function was passed through a Laplace cumulative distribution function in order to generate probabilities for reporting perceived rotation. *M*_*ES*_ from Eq (3), the mean, μ, and spread parameters β = σ √2, for the Laplace distribution, and a *shift* parameter that slides the function along the *x* axis were least-squares fit to the perceived rotation data across the "light luminance bars," "light contrast bars," and "dark bars" conditions for JJD, RSH, and WWS jointly (the “average luminance” condition was omitted during the fit since both the predicted and observed probabilities were essentially constant). Then, using *M*_*ES*_ = 7.79, $\hat{\mu }=0.480$, and *shift* = -0.096 from the joint least-squares fit, the spread, β, parameter was fit individually for JJD, RSH, and WWS across the "light luminance bars," "light contrast bars," and "dark bars" conditions. Our reasoning was that the spread of the psychometric functions would be expected to vary across subjects while, according to our model, the remaining three parameters would be specific to the particular experimental context, and thus common across the three subjects.

**Fig 7:**
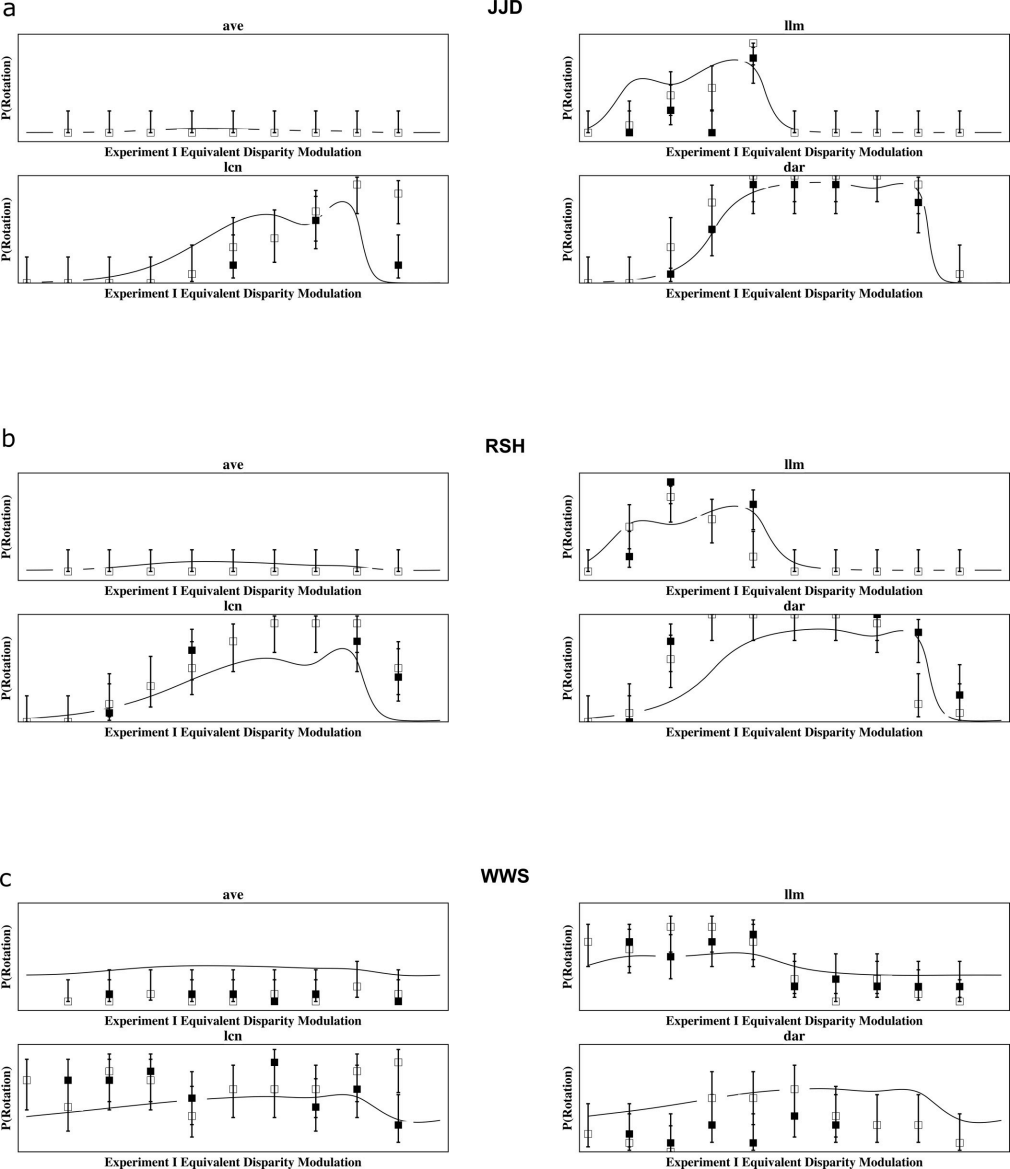
Model Predictions for perceived rotation. Predictions of the probability of perceiving rotation by the generalized difference model coupled with the presumed relationship of average luminance and contrast in controlling the response rate of neurons described by Eq (2) for participants (a) JJD, (b) RSH, and (c) WWS. The top left plot is for "average luminance" ("avg"), top right "light luminance bars" ("llm"), bottom left "light contrast bars" ("lcn"), and bottom right "dark bars" ("dar"). Probability of perceiving a rotation data are from the corresponding plots in Fig 6. Filled boxes are for the left "eye" condition and empty boxes for right "eye." Error bars indicate 95% confidence intervals based on the score estimator [60] (see also [61, 62]).

The plots in Fig 7 show the resulting fits for the three subjects across all four conditions (“average luminance” as well as the "light luminance bars," "light contrast bars," and "dark bars" conditions). Adjusted *R*^2^ ([66], p. 137; [67]) were calculated across all four conditions for JJD ($\hat{\sigma }=0.120$; adj-*R*^2^ = 0.824), RSH ($\hat{\sigma }=0.192$; adj-*R*^2^ = 0.762), and WWS ($\hat{\sigma }=1.29$; adj-*R*^2^ = 0.722). Of the 78 probabilities represented in each figure, the percentages of those with 95% confidence intervals that include the model prediction are 90% of the intervals for JJD, 79% of the intervals for RSH, and 64% of the intervals for WWS.

Clearly, the generalized difference model coupled with the presumed relationship of average luminance and contrast in controlling the response rate of neurons described by Eq (2) captures the results for perceived rotation, accounting for 72% to 82% of the variance in each participant’s responses. Little rotation is seen in the avg condition; rotation is perceived only with moderate Experiment I equivalent disparities in the llm condition; rotation is perceived over the upper range of disparities in the lcn condition; and rotation is perceived over a relatively wide range of disparities in the dar condition. The perceived rotation data from WWS, which differ from JJD and RSH in the llm, lcn, and dar conditions, require a larger spread parameter for the Laplace distribution than JJD or RSH, consistent with a perceptual bias to perceive rotation in one direction that was measured for this subject by Hetley and Stine (Appendix in [7]). The main conclusion, of course, is that perceived rotation can be well described as a function of the luminance or contrast disparity of the stimulus.

#### 2.3.5 Discussion

Straddling the background would seem to be necessary in order to see luster and/or rivalry with our stimuli, in accord with Anstis [32], Fry and Bartley [56], and Wolfe and Franzel's [31] observations on the occurrence of binocular luster and binocular rivalry, and Georgeson et al.’s model [38]. That threshold luminance disparities for the light luminance bars for perceiving luster or rivalry were close to those measured in Experiment I suggests that, for our displays, the luminance disparity of the bright bars drives most of the appearance of luster or rivalry (note that the dark bars simply never straddled the background in Experiment I). One possible exception is RSH, whose thresholds were below those from Experiment I, and slightly below the point where the luminance of the bright bars of the dichoptic grating pairs straddled that of the background. This may represent the impact of a personal criterion more than a physical threshold. However, data like those of RSH are also present in the literature, as Anstis [32] reported moderate ratings of luster when stimuli nearly straddled the background, which can be described by adding noise to the system [38]. In all cases, a larger disparity was required to perceive rivalry than luster.

Luster and rivalry arise in similar circumstances and co-vary. Hence, there may be a connection between luster and rivalry (first discussed with the observation that the two phenomena can be concurrent by Dove, as described by Helmholtz, [9], p. 514) in the form of a common, shared, underlying mechanism within the processing of binocular luminance and contrast information. The degree to which binocular luster and rivalry share a common mechanism, one would expect any theory of luster to be rather involved (see, for example, [53], Ch. 12).

With respect to the specific role of possible mechanisms invoked by straddling the background, imagine the image of a vertical edge separating a bright region on the left from a dark region on the right in the left retina and the opposite configuration in a corresponding location of the right retina. As suggested by Anstis [32], on-center ganglion cells in the left retina whose centers are to the left of the vertical edge will respond vigorously as will corresponding off-center cells in the right retina, and *vice versa* (cf., [68–71]). The resulting local anti-correlation that is driven by on-channel responses in correspondence with off-channel responses may contribute to the perception of luster at low disparities and rivalry at high disparities. Note that a rivalrous stereo pair without such a mixture of signals would contradict this local on/off theory.

That the appearance of luster and rivalry radically differ and that the threshold for rivalry is consistently higher than that for luster, of course, implies that the overlap between luster and rivalry is not complete; they involve distinct physiological states.

Perceived rotation, or the Venetian blind effect, followed generalized difference model predictions.

## 3. Conclusions

Our results, together with those of Hetley and Stine [7], expand our understanding of the processing of binocular luminance and contrast information. Hetley and Stine demonstrated for a fused image of a square-wave grating that perceived rotation varied as a function of the interocular difference in average luminance or contrast, while the brightness or perceived contrast varied with the interocular sum. Hence, distinct mechanisms control perceived rotation (the Venetian blind effect) versus brightness or perceived contrast for fused square-wave gratings presented with either average luminance or contrast disparities over and above the physiological states suggested by the different appearances of rotation versus the brightness or perceived contrast of the fused images (cf., [17]).

By constraining images to vary only in luminance or contrast, we are able to compare phenomena without interference by other perceptual processing, e.g., processing of geometric disparity to induce binocular rivalry. Experiments I and II demonstrate that luster and rivalry also vary with the interocular difference in average luminance or contrast. However, that luster and rivalry are tied to straddling the background luminance (consistent with a local on/off theory [32], and extending work by Anstis [32], Fry and Bartley [56], Wolfe and Franzel [31], and Georgeson et al. [38], while perceived rotation is captured by the generalized intensity difference model suggests that luster and rivalry, on the one hand, and perceived rotation, on the other, are also controlled by distinct mechanisms over and above the physiological states suggested by their distinct appearances.

Further, that luster and rivalry are perceptually distinct with the threshold for rivalry being higher than that for luster suggests that they represent distinct physiological states. They nonetheless share a link to straddling the background, perhaps consistent with Howard's [13] proposal that binocular luster is the result of brightness summation during mosaic dominance, with luster occurring during moments of transition (e.g., Dove, as described by Helmholtz [9], p. 525) or occurring when rivalry is halted (e.g., [9, 10]), and a local on/off theory [32].

## Supporting information

S1 Table Experiment I luminance modulation stimulus values. Listed is the average luminance viewed by each eye for the modulations used in Experiment I when the left eye’s image had the higher average luminance.
(PDF)

S2 Table Experiment I contrast modulation stimulus values. Listed is the luminance contrast viewed by each eye for the modulations used in Experiment I when the left eye’s image had the higher contrast.
(PDF)

S3 Table Experiment II avg stimulus values. Listed is the luminance of the individual bars viewed by each eye for the avg modulation used in experiment II when the left eye’s image had the higher luminance bar.
(PDF)

S4 Table Experiment II llm stimulus values. Listed is the luminance of the individual bars viewed by each eye for the llm modulation used in experiment II when the left eye’s image had the higher luminance bar.
(PDF)

S5 Table Experiment II lcn stimulus values. Listed is the luminance of the individual bars viewed by each eye for the lcn modulation used in experiment II when the left eye’s image had the higher luminance bar.
(PDF)

S6 Table Experiment II dar stimulus values. Listed is the luminance of the individual bars viewed by each eye for the dar modulation used in experiment II when the left eye’s image had the higher luminance bar.
(PDF)
