# Supplementary material for: At least two distinct mechanisms control binocular luster, rivalry, and perceived rotation with contrast and average luminance disparities
Source: PLoS One. 2019 May 21;14(5):e0215716. doi: 10.1371/journal.pone.0215716 (PMC6529001; doi:10.1371/journal.pone.0215716)
Supplement: S1 Table — Listed is the average luminance viewed by each eye for the modulations used in Experiment I when the left eye’s image had the higher average luminance. (PDF) [file pone.0215716.s001.pdf]

Luminance Modulation<br>Contrast = 0.5	Left Eye<br>Ave Luminance (cd/m^2)	Right Eye<br>Ave Luminance (cd/m^2)
0.0	42.5	42.5
0.1	46.8	38.3
0.2	51.0	34.0
0.3	55.3	29.8
0.4	59.5	25.5
0.5	63.8	21.3
0.6	68.0	17.0
0.7	72.3	12.8
0.8	76.5	8.5
0.9	80.7	4.2
