# Supplementary material for: At least two distinct mechanisms control binocular luster, rivalry, and perceived rotation with contrast and average luminance disparities
Source: PLoS One. 2019 May 21;14(5):e0215716. doi: 10.1371/journal.pone.0215716 (PMC6529001; doi:10.1371/journal.pone.0215716)
Supplement: S2 Table — Listed is the luminance contrast viewed by each eye for the modulations used in Experiment I when the left eye’s image had the higher contrast. (PDF) [file pone.0215716.s002.pdf]

Contrast Modulation<br>Average Luminance = 42.5 cd/m^2	Left Eye<br>Luminance Contrast	Right Eye<br>Luminance Contrast
0.00	0.50	0.50
0.10	0.55	0.45
0.20	0.60	0.40
0.30	0.65	0.35
0.40	0.70	0.30
0.50	0.75	0.25
0.60	0.80	0.20
0.70	0.85	0.15
0.80	0.90	0.10
0.90	0.95	0.05
