# Supplementary material for: At least two distinct mechanisms control binocular luster, rivalry, and perceived rotation with contrast and average luminance disparities
Source: PLoS One. 2019 May 21;14(5):e0215716. doi: 10.1371/journal.pone.0215716 (PMC6529001; doi:10.1371/journal.pone.0215716)
Supplement: S4 Table — Listed is the luminance of the individual bars viewed by each eye for the llm modulation used in experiment II when the left eye’s image had the higher luminance bar. (PDF) [file pone.0215716.s004.pdf]

Experiment I llm Equivalent Modulation	Left Eye Bar Luminance (cd/m^2)	Right Eye Bar Luminance (cd/m^2)
0.0	63.8	63.8
0.1	70.1	57.4
0.2	76.5	51.0
0.3	82.9	44.6
0.4	89.3	38.3
0.5	95.6	31.9
0.6	102.0	25.5
0.7	108.0	19.1
0.8	115.0	12.8
0.9	121.0	6.4
